# Adaptive Spatial–Temporal Aware Graph Learning for EEG-Based Emotion Recognition

**DOI:** 10.34133/cbsystems.0088

**Published:** 2024-05-08

**Authors:** Weishan Ye, Jiyuan Wang, Lin Chen, Lifei Dai, Zhe Sun, Zhen Liang

**Affiliations:** ^1^ School of Biomedical Engineering, Medical School, Shenzhen University, Shenzhen, China.; ^2^ Guangdong Provincial Key Laboratory of Biomedical Measurements and Ultrasound Imaging, Shenzhen, China.; ^3^ School of Clinical Medicine, Harbin Medical University, Harbin, China.; ^4^ Faculty of Health Data Science and Faculty of Medicine, Juntendo University, Tokyo, Japan.; ^5^ International Health Science Innovation Center, Medical School, Shenzhen University, Shenzhen, China.

## Abstract

An intelligent emotion recognition system based on electroencephalography (EEG) signals shows considerable potential in various domains such as healthcare, entertainment, and education, thanks to its portability, high temporal resolution, and real-time capabilities. However, the existing research in this field faces limitations stemming from the nonstationary nature and individual variability of EEG signals. In this study, we present a novel EEG emotion recognition model, named GraphEmotionNet, designed to enhance the accuracy of EEG-based emotion recognition through the incorporation of a spatiotemporal attention mechanism and transfer learning. The proposed GraphEmotionNet model can effectively learn the intrinsic connections between EEG channels and construct an adaptive graph. This graph’s adaptive nature is crucial in optimizing spatial–temporal graph convolutions, which in turn enhances spatial–temporal feature characterization and contributes to the process of emotion classification. Moreover, an integration of domain adaptation aligns the extracted features across different domains, further alleviating the impact of individual EEG variability. We evaluate the model performance on two benchmark databases, employing two types of cross-validation protocols: within-subject cross-validation and cross-subject cross-validation. The experimental results affirm the model’s efficacy in extracting EEG features linked to emotional semantics and demonstrate its promising performance in emotion recognition.

## Introduction

Affective computing represents a dynamic and burgeoning interdisciplinary domain that has drawn the attention of researchers hailing from diverse fields, such as computer science, neuroscience, psychology, and signal processing [1]. In particular, compared to other domains such as intelligent disease diagnosis, understanding the mechanism of the mind using biological information such as electroencephalography (EEG) signals and electromyography signals [2–4] could benefit the development of clinical treatment methods for psychiatric disease [5]. In recent years, there has been a growing emphasis on emotion recognition through the analysis of EEG signals, highlighting its escalating importance within the field of affective computing and human emotion analysis [6,7]. Emotion recognition from EEG signals is an important and challenging problem in the field of affective computing today. Understanding an individual’s emotional state is crucial for various applications including human–computer interaction, psychological health monitoring, and virtual reality experiences. EEG is a noninvasive physiological measurement capturing the spatial–temporal dynamics of brain activity. However, extracting emotional information from EEG is a challenging task due to its low spatial resolution and susceptibility to physiological and environmental interferences. Moreover, the interindividual differences in neural activity and the complexity of emotional experiences further increase the complexity of emotion recognition. Our research motivation is from a deep understanding of these challenges and the attention given to the major gaps in current EEG-based emotion recognition research. Despite some progress made, many existing methods still have limitations in dealing with noise, interindividual differences, and the dynamic performance of emotional expressions.

This study aims to address the following major research gaps: (a) Lack of comprehensive modeling of dynamic emotional experiences. Current EEG-based emotion recognition methods often overlook the temporal dynamics of emotional experiences. Our model aims to more comprehensively model dynamic emotional changes in order to improve recognition performance. (b) Insufficient modeling of interindividual differences. The differences in neural activity between individuals are crucial for emotion recognition, but few models can effectively capture these interindividual differences. Our approach aims to address this issue through adaptive graph learning. (c) Lack of modeling complex relationships between different emotions. The relationships between emotions are diverse and complex, but existing methods often oversimplify them. We introduce spatial–temporal attention mechanisms to better capture the complex spatial–temporal relationships between emotions. By addressing these key issues, we strive to advance the field of EEG-based emotion recognition, enhance the robustness and accuracy of models, and better serve the needs of practical applications. A well-crafted EEG-based model for emotion recognition carries the potential to drive progress in data processing, elevate the quality of discriminative feature representation, and ultimately elevate the overall performance of the model.

In recent years, there has been a notable surge in the adoption of deep learning techniques in EEG-based emotion recognition research. This is primarily attributed to their remarkable prowess in acquiring intricate feature representations. For example, convolutional neural networks (CNNs) and recurrent neural networks (RNNs) have emerged as prominent tools for deriving pertinent feature insights from either raw or transformed EEG data. For example, Zhang et al. [8] introduced a cascaded and parallel convolutional recursive neural network, demonstrating its effectiveness in acquiring distinctive EEG features. Niu et al. [9] developed a novel deep residual neural network integrating brain network analysis with a channel-spatial attention mechanism. However, these existing methods failed to consider the innate interconnections that exist between distinct brain regions during modeling.

Given the non-Euclidean spatial characteristics of brain regions, the adoption of a graph structure as a data representation is becoming increasingly favored for capturing the complex interrelationships among various brain channels. Inspired by the impressive achievements of graph convolutional network (GCN) models, researchers have delved into graph-based methodologies for EEG-based emotion recognition. In this paradigm, each EEG channel is equated to a node within the graph, and the interconnections between these channels are represented as the edges of the graph. Recently, graph-based EEG-based emotion recognition has been broadly classified into 2 main categories: spatial convolution and spectral convolution. Spatial convolution methods are characterized by their direct execution of convolution operations on the nodes and their adjacent nodes within the graph. For example, Duvenaud et al. [10] introduced a CNN structure that empowers end-to-end convolution operations on graphs. The GraphSAGE model [11] generated embedding vectors by sampling and aggregating features from a node’s local neighborhood. Additionally, graph attention networks [12] employed masked self-attention layers to allocate varying weights to different nodes within the graph, thereby enabling more nuanced and context-aware graph convolution operations. On the other hand, spectral convolution methods typically formulate convolution operations based on the spectral representation of the graph. For example, Bruna et al. [13] put forth a universal graph convolution framework that hinges on the graph Laplacian matrix. Defferrard et al. [14] employed Chebyshev expansion to mitigate computational complexity. Kipf and Welling [15] introduced a simplified GCN model tailored for semi-supervised learning. In the existing graph-based EEG-based emotion recognition methods, one of the main challenges revolves around the reliance on fixed and predetermined graph structures. Given the dynamic nature of emotional states, the utilization of a predefined graph structure often falls short of being sufficiently adaptive. Moreover, another main challenge is the identification of emotion transition rules. Generally, GCNs are directly applied to EEG-based emotion classification, overlooking the intricate dynamics of emotional transitions over time. In practical applications, an individual’s current emotional state is intimately connected to their preceding and subsequent emotional states. Hence, it becomes imperative to incorporate the understanding of emotional transition rules and delve into the temporal intricacies of emotions, ultimately enhancing the precision of emotion classification.

In order to tackle the primary challenges inherent in current graph-based EEG-based emotion recognition methods, we introduce GraphEmotionNet in this paper. GraphEmotionNet is a novel deep graph neural network explicitly developed for EEG-based emotion classification. It is designed to acquire an adaptive graph structure representation, optimally tailored to empower dynamic spatiotemporal graph convolution networks specifically for EEG emotion classification. The main contributions of this study are summarized below.

•We design a dynamic graph structure, devoid of predefinitions, allowing it to adapt in response to the inherent characteristics of the data distribution.

•We develop emotion-specific spatiotemporal convolutions, which encompass graph convolutions for capturing spatial features and time convolutions for discerning transitions between various EEG emotions.

•We integrate the feature alignment between the source domain and target domain into our modeling process, effectively mitigating the impact of individual EEG variability on the model’s performance.

## Methods

In our study, GraphEmotionNet is defined as an undirected graph, given as *G* = (*V*, *E*, *A*). *V* represents the set of vertices, where each vertex corresponds to an electrode on the brain. The number of vertices ∣*V*∣ is equal to the total *N* electrodes. *E* indicates the set of edges, symbolizing the connections that link the vertices within the graph. *A* denotes the adjacency matrix. In contrast to the conventional approach of calculating *A*, the proposed GraphEmotionNet dynamically learns the values of *A* during the model training process.

**Fig. 1.**
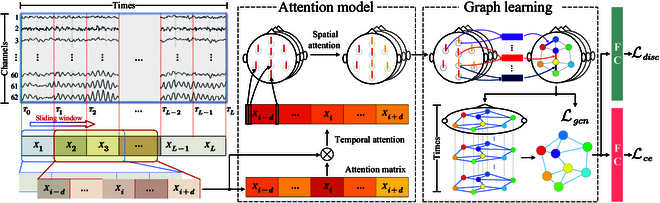
An overview of the proposed GraphEmotionNet.

As shown in Fig. 1, a sequence of one EEG trial denotes as $X=\left(X_{1},X_{2},\ldots ,X_{L}\right)\in {\mathbb{R}}^{L\times N\times F_{\mathrm{de}}}$. Here, *L* corresponds to the total number of EEG samples within the trial. Each individual EEG sample *X_i_* has a duration of 1 s and is characterized by differential entropy (DE) features [16], which is denoted as $X_{i}=\left(x_{1}^{i},,,x_{2}^{i},,,\ldots ,,,x_{N}^{i}\right)\in {\mathbb{R}}^{N\times F_{\mathrm{de}}}$. Here, $x_{n}^{i}\in {\mathbb{R}}^{F_{\mathrm{de}}}$, where *n* is in the range of 1, 2, …, *N*.

To enable the model to learn emotion-specific temporal features, we define the model input as $\begin{array}{c} X_{i}=\left(X_{i-d},,,\ldots X_{i},,,\ldots ,,,X_{i+d}\right) \end{array}\begin{array}{c} \in {\mathbb{R}}^{T\times N\times F_{\mathrm{de}}} \end{array}$, *d* ∈ ℕ^+^ represents the temporal context coefficient, and *T* = 2*d* + 1 indicates the data length. Then, for each single trial *X*, a total of *L* − 2*d* input data could be constructed.

### Spatial–temporal attention

To capture valuable spatiotemporal information, a fused spatiotemporal attention model is introduced, which is composed of temporal attention extraction and spatial attention extraction.

#### Temporal attention extraction

For the temporal information encoded in EEG signals, we observe that there exists a noteworthy correlation between 2 consecutive data samples denoted as *X_i_* and *X_j_* (1 ≤ *i* ≠ *j* ≤ *L*). This correlation exhibits the capacity to dynamically adjust to diverse emotional states. To enhance the precision of temporal information capture, we introduce a temporal attention mechanism on the input data X, allowing for the dynamic extraction of temporal patterns associated with brainwave emotions.

The definition of the temporal attention mechanism is given as:

$$Q=V_{e}\cdot \sigma \left(\left(X\prime U_{1}\right)U_{2}\left(U_{3}X^{\prime \prime }\right)+b_{e}\right),$$ (1)

where *V_e_* and *b_e_* ∈ ℝ^*T*×*T*^. *U*_1_ ∈ *ℝ^N^*, *U*_2_ ∈ *ℝ*^*F_de_*× *N*^, and *U*_3_ ∈ *ℝ^F_de_^* are learnable parameters. $X\prime \in {\mathbb{R}}^{T\times F_{\mathrm{de}}\times N}$, and $X^{\prime \prime }\in {\mathbb{R}}^{N\times F_{\mathrm{de}}\times T}$. *σ* represents the sigmoid activation function, and it computes the temporal attention matrix *Q* ∈ *ℝ*^*T*×*T*^.

$$Q_{m,n}^{\prime }=\text{softmax}\left(Q_{m,n}\right)=\frac{\text{exp}\left(Q_{m,n}\right)}{\sum_{n=1}^{T}‍\text{exp}\left(Q_{m,n}\right)},$$ (2)

*Q*_*m*, *n*_ denotes the temporal correlation between the samples *X_m_* and *X_n_* (*X_m_* and *X_n_* are from X). Finally, the temporal attention matrix *Q* is normalized through a softmax operation as shown in Eq. 2.

Then, the model input could be adjusted through the obtained temporal attention as:

$$\hat{X}=\;\;(X_{1},\;X_{2},\;...\;,\;X_{T})Q\prime ,$$ (3)

where $\begin{array}{c} \hat{X}=({\hat{X}}_{1},\;{\hat{X}}_{2},\;...\;,\;{\hat{X}}_{T})\in {\mathbb{R}}^{T\times N\times F_{\mathrm{de}}} \end{array}$ could focus more on valuable temporal information.

#### Spatial attention extraction

Besides, the spatial information embedded in EEG signals underscores the unique roles that various brain regions play in shaping the array of emotions individuals experience. To elevate the precision of spatial information capture, we introduce a spatial attention mechanism designed to automatically extract spatial attention dynamics, given as:

$$P=V_{s}\cdot \sigma \left(\left({\hat{X}}^{\prime \prime }W_{1}\right)W_{2}{\left(W_{3}\hat{X}\prime \right)}^{T}+b_{s}\right),$$ (4)

where *V_s_* and *b_s_* ∈ *ℝ*^*N*×*N*^. $W_{1}\in {\mathbb{R}}^{T}$, $W_{2}\in {\mathbb{R}}^{F_{\mathrm{de}}\times T}$, and $W_{3}\in {\mathbb{R}}^{F_{de}}$ are learnable parameters. $\hat{X}\prime \in {\mathbb{R}}^{T\times F_{\mathrm{de}}\times N}$, and ${\hat{X}}^{\prime \prime }\in {\mathbb{R}}^{N\times F_{\mathrm{de}}\times T}$. The computed *P* ∈ ℝ^*N*×*N*^ indicates the spatial attention matrix. *P* is further normalized through a softmax operation as:

$$P_{m,n}^{\prime }=\text{softmax}\left(P_{m,n}\right)=\frac{\text{exp}\left(P_{m,n}\right)}{\sum_{n=1}^{N}‍\text{exp}\left(P_{m,n}\right)},$$ (5)

$P_{m,n}^{\prime }$ denotes the normalized spatial correlation between the EEG channels *m* and *n*. In the present study, the spatial attention matrix *P*^′^ undergoes dynamic updates in conjunction with the node representation updates, as detailed in the “Adaptive graph learning” section.

### Adaptive graph learning

In order to adaptively and autonomously learn node representations by taking into account the interconnections and topological structure among EEG channels and to bolster the effectiveness of graph-based representation, we introduce an adaptive graph learning method. This method is designed to capture the intrinsic relationships among EEG channels, optimizing their contribution to the spatial–temporal GCN (“Spatial–temporal graph convolution” section) for improved emotion classification.

To capture the dynamic relationships between EEG channels, we employ an adaptive graph learning approach. Firstly, we initialize the adjacency matrix where the elements represent the connectivity strength between different EEG channels. This initialization is based on the spatial distance between the channels. Then, during the training process, we use the backpropagation algorithm to update the adjacency matrix. The dynamic updating of the adjacency matrix is based on the evaluation of the current task performance by our model and its fit to the actual scenario. This updating process aims to make the graph structure better adapt to the specific features of the current emotion recognition task while being closer to the true underlying connections between EEG channels. We adjust the adjacency matrix through the optimization process, minimizing the loss function while preserving the topological structure of the graph as much as possible. This helps the model to flexibly capture and express the correlations between channels. This adaptive optimization strategy makes our model more adaptable and generalizable, aiding in capturing the dynamic features of different emotional states more accurately. In the present study, the dynamic updating of the adjacency matrix allows the graph structure to be adjusted according to the task requirements. The weights in the graph structure reflect the relative importance between channels, which helps the model to more accurately capture the relevant information for emotional expression. The adaptive graph learning method not only improves the model’s representation capability of EEG signals but also enables it to adapt more flexibly to different emotion recognition tasks. During the process of training optimization, the objective function includes minimizing the loss function and maximizing the representation capability of the graph. We achieve this through weighted graph Laplacian regularization. We employ stochastic gradient descent or other optimization algorithms to minimize the objective function and update the adjacency matrix in each training iteration. In the presented adaptive graph learning method, we designate EEG channels as the nodes within the graph. Based on the input feature matrix $X_{i}=\left(x_{1}^{i},,,x_{2}^{i},,,\ldots ,,,x_{N}^{i}\right)\in {\mathbb{R}}^{N\times F_{\mathrm{de}}}$, we introduce an adjacency matrix *A_mn_* = *g*(*x_m_*, *x_n_*) to delineate the connections between nodes *x_m_* and *x_n_*. Unlike traditional adjacency matrices, which are typically constructed based on prior knowledge (e.g., k-nearest neighbor graphs), the defined *A_mn_* is adaptively learned through a neural network layer with a learnable weight vector $w=\left(w_{1},,,w_{2},,,\ldots ,,,w_{F_{de}}\right)\in {\mathbb{R}}^{F_{de}\times 1}$. The definition of *A_mn_* is given as

$$A_{\mathrm{mn}}=\frac{\text{exp}\left(-\text{ReLU}\left(w^{T}\left|x_{m}-x_{n}\right|\right)\right)}{\sum_{n=1}^{N}‍\text{exp}\left(-\text{ReLU}\left(w^{T}\left|x_{m}-x_{n}\right|\right)\right)}.$$ (6)

The inclusion of the linear rectification function (ReLU) serves to maintain nonnegativity in the distances between nodes when they are multiplied by the weight vector ***w***. The learnable weight vector *w* is updated by minimizing the following loss function:

$$L_{\text{gcn}}=\tau \sum_{m,n=1}^{N}{∥x_{m}-x_{n}∥}_{2}^{2}A_{\mathrm{mn}}+{∥A∥}_{F}^{2}$$ (7)

A smaller value of *A_mn_* signifies a greater Euclidean distance between the nodes *x_m_* and *x_n_*. Given that the brain connectivity structure is not a fully connected graph, we incorporate the second term $∥A{∥}_{F}^{2}$ to regulate the sparsity of the graph represented by matrix *A*. Here, *τ* ≥ 0 serves as the regularization parameter.

### Spatial–temporal graph convolution

In the present study, each time segment data is depicted as a graph structure, as indicated by the learned adjacency matrix introduced in the “Adaptive graph learning” section. A spatial–temporal graph convolution is designed to integrate GCNs with spatiotemporal data, enabling the modeling of spatiotemporal relationships and the extraction of spatial–temporal features. Here, spatial–temporal graph convolution updates node feature representations by considering the node’s neighbors and changes in the spatiotemporal dimensions. This operation effectively harnesses the interplay between the graph’s structure and the spatiotemporal information embedded within EEG signals to acquire spatiotemporal feature representations for individual nodes.

#### Spatial graph convolution

Spatial graph convolution updates node feature representations by aggregating the features of neighboring nodes. Unlike traditional CNNs that are primarily used for grid-like structured data, spatial graph convolution can be applied to any graph structure. This makes it a flexible and adaptable approach for various data types that exhibit nongrid, network-like structures.

Specifically, spatial graph convolution leverages the learned adjacency matrix *A* to establish relational weights between nodes, conducting aggregation operations on neighboring node features. To extract spatial features within the spatial dimension, we employ graph convolution based on spectral graph theory. The Chebyshev expansion of the graph Laplacian is employed to effectively reduce computational complexity. The Chebyshev graph convolution of a (*K*−1)th order polynomial is defined as follows:

$$g_{\theta }{*}_{G}x=g_{\theta }\left(L\right)x=\sum_{k=0}^{K-1}\theta_{k}\xi_{k}\left(\tilde{L}\right)x$$ (8)

where *g_θ_* represents the convolutional kernel, ∗*_G_* denotes the graph convolution operation, *θ* ∈ *ℝ^K^* represents the Chebyshev coefficient vector, and *x* is the input data. *L* = *D* − *A* is the Laplacian matrix, with the degree matrix of *D* ∈ *ℝ*^*N*×*N*^.

$$\tilde{L}=\frac{2}{\lambda_{\max}}L-I_{N},$$ (9)

where *λ_max_* is the maximum eigenvalue of the Laplacian matrix, and *I_N_* is the identity matrix. The expression *ξ_k_*(*x*) = 2*xξ*_*k*−1_(*x*) − *ξ*_*k*−2_(*x*) represents the recursive Chebyshev polynomial, with initial conditions *ξ*_0_(*x*) = 1 and *ξ*_1_(*x*) = *x*. By employing the Chebyshev polynomial’s approximation expansion, information is gathered from neighbors up to the (*K*−1)th order for each node, using the node itself as the central reference. This enables the model to capture information from a local neighborhood of nodes up to *K*−1 hops away from the central node. Subsequently, these polynomials are used in the Chebyshev graph convolution operation to approximate the spectral filtering of graph signals.

In our work, we extend the above definitions to graphs with multiple EEG channel nodes. Equation 8 can then be rewritten as:

$${\hat{X}}_{\mathrm{sgc}}=\sum_{t=0}^{T}\sum_{k=0}^{K-1}\theta_{k}\xi_{k}\left(\tilde{L}\right)P_{t}^{\prime }{\hat{X}}_{t}$$ (10)

${\hat{X}}_{t}\in {\mathbb{R}}^{N\times F_{\mathrm{de}}}$ is *t*th time segment data, and $P_{t}^{\prime }$ is the learned spatial attention matrix in the “Spatial attention extraction” section. *θ_k_* is a learnable parameter. ${\hat{X}}_{\mathrm{sgc}}\in {\mathbb{R}}^{N\times F_{\text{out}}}$, and *F_out_* is the output dimension of the spatial graph convolution. Through multiple layers of spatial graph convolution operations, node representations are gradually refined. This iterative process enhances the extraction of higher-level spatial features and a better capture of spatial correlations and patterns between nodes.

#### Temporal graph convolution

To capture emotional transition rules, we integrate temporal graph convolution operations into our approach. In this process, based on the learned adjacency matrix, the temporal graph convolution is applied to nodes in chronological order. In particular, after extracting spatial features from each time segment data through spatial graph convolution operations, we implement a standard 2D convolutional layer to capture the temporal context information. The temporal convolution operation is defined as follows:

$${\hat{X}}_{\mathrm{tgc}}=\text{ReLU}\left(\Phi *\left(\text{ReLU}\left({\hat{X}}_{\mathrm{sgc}}\right)\right)\right).$$ (11)

Here, ReLU serves as the activation function, Φ represents the parameters of the convolutional kernel, and ∗ denotes the standard convolution operation.

Throughout this temporal convolution process, we could consider the impact of neighboring nodes’ features and transition rules, allowing us to capture patterns in emotional state transitions. These learned rules play a critical role in combining adjacent emotional categories, ultimately contributing to the classification of the current emotional category.

### Domain adaptation

Domain adaptation is frequently employed to tackle variations arising from factors like intersubject variability, task specificity, domain shift, and model stability. It serves the purpose of addressing discrepancies between different domains and improving model generalization [17–20]. In the study, we define the source domain and target domain as S and T and incorporate domain adversarial neural networks (DANNs) for feature alignment.

Specifically, domain adversarial training minimizes the domain discrepancy between $f\left({\hat{X}}_{\mathrm{tgc}}^{S}\right)$ (source domain sample features) and $f\left({\hat{X}}_{\mathrm{tgc}}^{T}\right)$ (target domain sample features). Here, *f*(·) is a feature extractor with parameters *θ_f_*, and *d*(·) is a discriminator with parameters *θ_d_*, *d*(·) is to distinguish whether the characterized sample features $f\left({\hat{X}}_{\mathrm{tgc}}^{S}\right)$ or $f\left({\hat{X}}_{\mathrm{tgc}}^{T}\right)$ originate from the source domain (S) or the target domain (T). The corresponding loss function is given as:

$$\begin{array}{c} \begin{array}{l} L_{\text{disc}}(\theta_{f},\theta_{d})=-\sum_{i=1}^{N_{s}}‍\text{log}d\left(f\left({\hat{X}}_{\mathrm{tgc}\_i}^{S}\right)\right)\\ -\sum_{i=1}^{N_{t}}‍\text{log}\left(1-d\left(f\left({\hat{X}}_{\mathrm{tgc}\_i}^{T}\right)\right)\right). \end{array} \end{array}$$ (12)

During the training process, we employ an end-to-end training approach, as described in [21], and achieve domain adversarial training by introducing a gradient reversal layer. The overall loss function for the final model is defined as:

$$\underset{\theta_{f}}{\text{min}}\underset{\theta_{d}}{\text{max}}\;L_{\mathrm{classifier}}(\theta_{f})+L_{\mathrm{gcn}}(\theta_{f})-\lambda L_{\mathrm{disc}}(\theta_{f},\theta_{d}),$$ (13)

where $L_{\text{classifier}}\left(\theta_{f}\right)$ represents the classification loss to measure the classification capabilities in the source domain. $L_{\text{gcn}}\left(\theta_{f}\right)$ is the adaptive graph learning loss introduced in the “Adaptive graph learning” section. $L_{\text{disc}}(\theta_{f},\theta_{d})$ is the adversarial loss to train the discriminator to distinguish features from the source and target domains. *θ_f_* and *θ_d_* are the parameters of *f*(·) and *d*(·). *λ* is the balance hyperparameter to ensure the stability of domain adversarial training, which is determined through an exponential growth approach, as follows:

$$\lambda =\frac{2}{1-\text{exp}(\;-\;p)}-1.$$ (14)

Here, *p* is a factor related to training epochs and is determined by the ratio of the current training epoch to the maximum training epochs. During the initial training phase, a larger value of *λ* emphasizes the domain classification loss, promoting the performance of the domain classifier. This helps the model to focus more on domain adaptation. In the late training stage, *λ* gradually decreases, allowing the feature extractor for domain classification to learn more flexible and robust features, consequently improving the performance of the task classifier. This dynamic adjustment strategy reflects a fine understanding of the different requirements at different stages of the model training process.

## Results and Discussion

### Benchmark databases

To assess the effectiveness of the proposed model, we conduct experiments on 2 publicly available EEG emotion databases: the SEED (SJTU Emotion EEG Dataset) database [22] and the SEED-IV database [23]. In the SEED database, 15 movie clips were used to induce 3 emotions (negative, neutral, and positive), and EEG signals were simultaneously recorded using a 62-channel ESI (Electric Source Imaging) Neuroscan system from 15 subjects in different emotional states (7 males and 8 females). In the SEED-IV database, 24 movie clips were used to induce 4 emotions (happy, sad, fear, and neutral), and EEG signals were recorded from 15 subjects in different emotional states (7 males and 8 females) using the same 62-channel ESI Neuroscan system. Each subject participated in 3 different experiments, each consisting of 24 different movie clips. As in the previous literature, we conducted the experiment with the first single session in SEED and with all 3 sessions in SEED-IV. This choice was made for considerations of data size and experimental design. Additionally, to have a more comprehensive evaluation of the model’s performance on SEED-IV, we also conducted experiments using a single session to ensure a thorough understanding of the model’s performance even with a smaller amount of data.

To ensure a fair comparison with other studies on the 2 benchmark databases, we also used the pre-computed DE feature data. A time context coefficient is set to *d*.

### Implementation details and model setting

In the experiments, the hyperparameter settings and optimization details are adopted as below. The context coefficient *d* is set to 3, which controls the information propagation range in the GCN. The order of Chebyshev polynomials *K* is set to 2, influencing the order up to which each node collects information from its neighboring nodes. The output feature dimension *F_out_* of the spatial GCN is set to 5, determining the final feature dimension for each node. The regularization parameter *tau* is set to 1 × 10^−2^, serving as a coefficient for regularization to control the model’s complexity and prevent overfitting. Both the feature extractor *f*(·) and the domain discriminator *d*(·) consist of fully connected layers. Specifically, *f*(·) is designed with a structure of 310-64-64-64, while *d*(·) is designed with a structure of 64-64-64-2/3, with the final layer using a Softmax activation function. The optimization is performed using the RMSprop optimizer with a learning rate set to 1 × 10^−3^, adjusting model parameters for better data fitting. The batch size is set to 96, indicating the size of the data batch used for training in each iteration. All models are trained on an NVIDIA GeForce RTX 1080 GPU using the PyTorch API with CUDA version 11.7. During the model training process, only the raw target data are used, and no label information is utilized. This approach aligns with previous EEG-based emotion recognition methods, employing a transfer learning framework, as demonstrated in prior studies [24–27]. These parameter settings and configurations are derived empirically to achieve optimal performance for the given task. Different tasks and databases may require adjustments to hyperparameters based on specific circumstances.

### Within-subject cross-validation experimental results

**Fig. 2.**
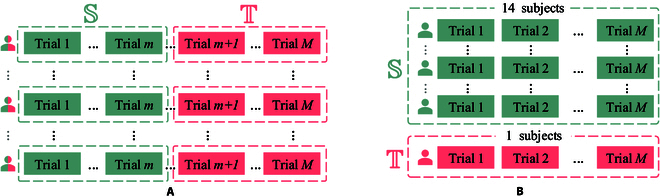
Cross-validation protocols in the experiments. (A) Within-subject cross-validation. For each subject, the first *m* trials are the source domain, and the remaining 15-*m* trials are the target domain. (B) Cross-subject cross-validation. One subject is the target domain, and the remaining 14 subjects are the source domain.

To assess the model’s performance on data obtained from the same subject, we conduct within-subject cross-validation (Fig. 2B), following a cross-validation protocol consistent with prior studies [17,19,25,28,29]. In the SEED database, for each subject, we use the first 9 trials as the source domain and the remaining 6 trials as the target domain. In the SEED-IV database, for each subject, we use the first 16 trials from all 3 sessions as the source domain and the remaining 8 trials from all 3 sessions as the target domain. The corresponding experimental results for within-subject cross-validation in the SEED database and SEED-IV database are presented in Tables 1 and 2, respectively. It shows that the proposed GraphEmotionNet model achieves good performance on both benchmark databases, when compared to existing methods. The GraphEmotionNet model obtains a 3-class classification accuracy of 94.68±9.07 on the SEED database and a 4-class classification accuracy of 71.62±7.49 on the SEED-IV database.

**Table 1.** Model performance on the SEED database using within-subject cross-validation

Methods	*P_acc_* (%)	Methods	*P_acc_* (%)
Traditional machine learning methods			
SVM [29]	83.99 ± 09.72	GRSLR [31]	87.39 ± 08.64
RF [29]	78.46 ± 11.77	GSCCA [32]	82.96 ± 09.95
CCA [29]	77.63 ± 13.21	DBN [22]	86.08 ± 08.34
Deep learning methods			
DGCNN [28]	90.40 ± 08.49	BiHDM [29]	93.12 ± 06.06
R2G- STNN [19]	93.38 ± 05.96	SimNet* [33]	90.13 ± 10.84
BiDANN [17]	92.38 ± 07.04	STRNN [34]	89.50 ± 07.63
GCNN [29]	87.40 ± 09.20	DANN [29]	91.36 ± 08.30
**GraphEmotionNet**			**94.68 ±** **09.07**

**Table 2.** Model performance on the SEED-IV database using within-subject cross-validation

Methods	*P_acc_* (%)	Methods	*P_acc_* (%)
Traditional machine learning methods			
RF* [35]	60.27 ± 16.36	KNN* [36]	54.18 ± 16.28
TCA* [37]	51.88 ± 15.84	CORAL* [38]	66.06 ± 15.13
SA* [39]	52.81 ± 09.53	GFK* [40]	56.14 ± 12.15
Deep learning methods			
DCORAL [41]	65.10 ± 13.20	DAN [41]	60.20 ± 10.20
DDC [41]	68.80 ± 16.60	DGCNN [28]	69.88 ± 16.29
**GraphEmotionNet**			**71.62** **± 07**.**49**

### Cross-subject cross-validation experimental results

Cross-subject leave-one-subject-out cross-validation stands as the prevailing evaluation protocol in current affective brain–computer interface systems. This cross-validation protocol provides an objective measure for assessing the model’s generalizability when considering data from different subjects. Specifically, we treat all data of one subject as the target domain and use all data of the remaining subjects as the source domain. We repeat the training and validation process until each subject’s data is considered as the target domain once. In Table 3, we present the cross-subject cross-validation experimental results on the SEED database, under a comparison with the literature. The results show that the proposed GraphEmotionNet model achieves a promising performance (86.43±6.66), with enhanced stability (lower variance value). The cross-subject cross-validation experimental results on the SEED-IV database are reported in Table 4. The corresponding classification accuracies on different subjects are 63.07±8.53. From the above experimental results, it is observed that our proposed GraphEmotionNet demonstrates notable performance improvements compared to other graph-based EEG emotion recognition methods, for example, DGCNN [28], which utilized a fixed graph structure to represent the spatial information of EEG signals. In contrast, our proposed model utilizes an adaptive graph learning method to dynamically construct the graph structure. This allows for a better capture of the spatial correlations in EEG signals. In addition, the DGCNN model did not take into account the temporal information of EEG signals, while the proposed model in this paper utilizes spatial–temporal attention mechanisms and spatial–temporal graph convolutions to extract both spatial and temporal features from EEG signals. This enables a more effective capture of the patterns of emotional state changes. Furthermore, the DGCNN model did not consider individual differences in EEG signals, while the proposed model in this paper utilizes domain adaptation methods to align the feature representations of different individuals. This enhances the model’s ability to generalize and adapt to individual variations in EEG signals.

**Table 3.** Model performance on the SEED database using cross-subject cross-validation

Methods	*P_acc_* (%)	Methods	*P_acc_* (%)
Traditional machine learning methods			
TKL [17]	63.54 ± 15.47	T-SVM [17]	72.53 ± 14.00
TCA [29]	63.64 ± 14.88	TPT [42]	75.17 ± 12.83
KPCA [42]	61.28 ± 14.62	GFK [29]	71.31 ± 14.09
SA [29]	69.00 ± 10.89	DICA [43]	69.40 ± 07.80
DNN [42]	61.01 ± 12.38	SVM [42]	58.18 ± 13.85
Deep learning methods			
DGCNN [28]	79.95 ± 09.02	DAN [42]	83.81 ± 08.56
BiHDM [29]	85.40 ± 07.53	MMD [44]	80.88 ± 10.10
R2G-STNN [19]	84.16 ± 07.63	SimNet* [33]	81.58 ± 05.11
BiDANN [17]	83.28 ± 09.60	DResNet [43]	85.30 ± 08.00
ADA [44]	84.47 ± 10.65	DANN [44]	81.65 ± 09.92
**GraphEmotionNet**			**86.43 ±** **06.66**

**Table 4.** Model performance on the SEED-IV database using cross-subject cross-validation

Methods	*P_acc_* (%)	Methods	*P_acc_* (%)
Traditional machine learning methods			
RF* [35]	50.98 ± 09.20	KNN* [36]	40.83 ± 07.28
SVM [42]	51.78 ± 12.85	Adaboost* [45]	53.44 ± 09.12
TCA [29]	56.56 ± 13.77	CORAL* [38]	49.44 ± 09.09
SA [29]	64.44 ± 09.46	GFK* [40]	45.89 ± 08.27
KPCA [42]	51.76 ± 12.89	DNN [42]	49.35 ± 09.74
Deep learning methods			
DGCNN [28]	52.82 ± 09.23	DAN [42]	58.87 ± 08.13
A-LSTM [46]	55.03 ± 09.28	DANN [42]	54.63 ± 08.03
**GraphEmotionNet**			**63.07 ±** **08.53**

In general, the differences between the proposed model and traditional graph-based models such as DGCNN, in terms of graph construction, feature extraction, and classification performance, can be summarized as follows: (a) Graph Construction. The proposed model uses a neural network layer to learn the adjacency matrix dynamically, thus constructing the graph structure adaptively. In contrast, DGCNN typically uses predefined adjacency matrices, such as k-nearest neighbor graphs or brain region-based graphs. (b) Feature Extraction. The proposed model utilizes spatial–temporal attention mechanisms and spatial–temporal graph convolutions to extract both spatial and temporal features from EEG signals. On the other hand, DGCNN only considers spatial graph convolutions to extract spatial features from EEG signals. (c) Classification Performance. The proposed model achieves better classification accuracy than DGCNN on both of the 2 benchmark databases, indicating its superior emotion recognition capability.

### Ablation study

We perform ablation experiments to gauge the influence and contributions of different modules in the proposed GraphEmotionNet model. Two distinct cross-validation protocols are employed for this evaluation. Tables 5 and 6 show the ablation results conducted using the within-subject cross-validation. Tables 7 and 8 show the ablation results conducted using the cross-subject cross-validation. The experimental results were validated on 2 benchmark databases (the SEED database for one session and the SEED-IV database for one session and all sessions), respectively.

**Table 5.** The ablation study of the proposed model using within-subject cross-validation

Methods	SEED	SEED-IV
Without discriminator	87.61 ± 08.50	71.04 ± 08.72
With fixed adjacency matrix (KNN)	94.32 ± 08.42	71.09 ± 09.39
Without temporal attention	93.87 ± 10.21	68.84 ± 08.96
Without spatial attention	93.81 ± 10.51	71.11 ± 06.81
Without temporal and spatial attention	92.32 ± 11.36	70.79 ± 07.06
**GraphEmotionNet**	**94.68 ±** **09.07**	**71.62 ** **± 07** **.49**

**Table 6.** The ablation study of the proposed model using within-subject cross-validation on 3 sessions of SEED-IV, respectively

Methods	Session 1	Session 2	Session 3
Without discriminator	81.88 ± 13.16	85.36 ± 11.87	92.00 ± 07.92
With fixed adjacency matrix (KNN)	80.47 ± 09.48	85.75 ± 11.79	91.40 ± 07.06
Without temporal attention	80.26 ± 11.89	85.36 ± 11.87	92.15 ± 08.03
Without spatial attention	81.67 ± 10.66	85.65 ± 09.33	92.40 ± 08.12
Without temporal and spatial attention	79.22 ± 15.26	84.11 ± 10.30	90.68 ± 08.97
**GraphEmotionNet**	**82.24 ± 11.00**	**85.94 ± 11.50**	**93.07 ±** **09.97**

**Table 7.** The ablation study of the proposed model using cross-subject cross-validation

Methods	SEED	SEED-IV
Without discriminator	78.53 ± 06.44	58.75 ± 08.62
With fixed adjacency matrix (KNN)	86.25 ± 06.03	62.90 ± 08.59
Without temporal attention	86.20 ± 07.03	62.76 ± 07.96
Without spatial attention	85.69 ± 06.09	62.77 ± 07.85
Without temporal and spatial attention	85.63 ± 03.94	61.94 ± 08.01
**GraphEmotionNet**	**86.43 ± ** **06.66**	**63.07 ±** **08.53**

**Table 8.** The ablation study of the proposed model using cross-subject cross-validation on 3 sessions of SEED-IV, respectively

Methods	Session 1	Session 2	Session 3
Without discriminator	62.03 ± 10.46	64.31 ± 09.99	66.71 ± 11.68
With fixed adjacency matrix (KNN)	64.90 ± 10.60	70.00 ± 13.05	73.68 ± 14.55
Without temporal attention	63.40 ± 10.94	69.57 ± 08.61	73.09 ± 11.62
Without spatial attention	64.15 ± 09.54	69.95 ± 06.87	69.57 ± 13.86
Without temporal and spatial attention	59.27 ± 09.04	69.43 ± 11.23	65.96 ± 15.56
**GraphEmotionNet**	**65.15 ± 10.81**	**70.24 ± 11.46**	**74.26 ± 15.72**

Here, we consider the proposed GraphEmotionNet model to omit a specific component as: the model without a discriminator (omitting domain adaptation), the model with a fixed adjacency matrix using KNN (excluding adaptive graph learning), the model without temporal attention (omitting the temporal-based attention mechanism), the model without spatial attention (excluding the spatial-based attention mechanism), and the model without both temporal and spatial attention (omitting both the temporal- and spatial-based attention mechanisms). The results highlight that the removal of any of these modules in the proposed model leads to a noticeable decline in emotion recognition performance, characterized by lower classification accuracy and higher variance values.

### Hyperparameter effect of *d* and *K*

**Fig. 3.**
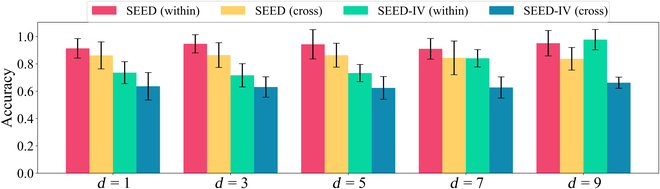
The hyperparameter effect of *d* on model performance under within-subject cross-validation (within) and cross-subject cross-validation (cross).

**Fig. 4.**
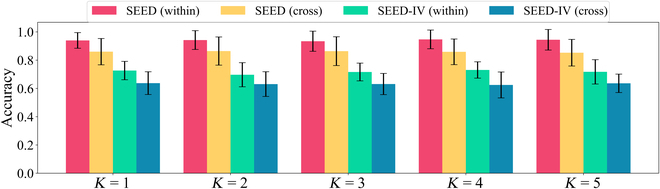
The hyperparameter effect of *K* on model performance under within-subject cross-validation (within) and cross-subject cross-validation (cross).

To comprehensively evaluate the stability of the proposed GraphEmotionNet model under different conditions, we further explore the impact of different hyperparameter configurations. In the analysis, we consider the input time segment data and vary the time coefficient parameter *d* across different values, specifically setting it to 1, 3, 5, 7, and 9, and assess the associated impact on model performance. The evaluation results are shown in Fig. 3. Additionally, we conduct an examination of the *K* value within the spatial–temporal convolution network, considering a range from 1 to 5. The corresponding results are presented in Fig. 4. Both types of parameter analyses indicate that the model’s performance remains remarkably stable across diverse parameter settings. The setting of the context coefficient *d* as an odd number (1, 3, 5, 7, and 9) ensures that the model can effectively utilize information from the previous and subsequent time steps. This odd selection helps the model to better capture and understand the temporal dynamics of the dynamic emotional changes. The Chebyshev polynomial order *K* is limited to integers from 1 to 5 to balance the accuracy of feature extraction and the robustness to potential noise. This design avoids introducing too much noise with excessively high orders, helping the model to extract key features better. In essence, the proposed GraphEmotionNet exhibits excellent generalizability when applied to different databases and under various cross-validation protocols, with limited dependence on specific parameter values.

### Validation on real-world clinical data

To further validate the adaptability of our model across various downstream tasks, we expanded the application of GraphEmotionNet on a real-world clinical EEG dataset. This dataset was obtained from the Hospital Universiti Sains Malaysia, featuring patients diagnosed with depression [30]. This dataset includes 27 healthy subjects (38.28 ± 15.64 years old) and 29 individuals diagnosed with major depressive disorder (MDD) (40.33 ± 12.86 years old). Their categorization aligns with the international diagnostic criteria for depression as per the *Diagnostic and Statistical Manual of Mnual Disorders, Fourth Edition*. The EEG data were collected using the Brain Master Discovery amplifier, employing a setup consisting of 19 scalp electrodes, at a sampling rate of 256 Hz. In the preprocessing, bandpass filtering ranging from 0.5 to 70 Hz and a notch filter at 50 Hz were conducted. The experimental protocol involved recording EEG signals from participants during both closed-eye and open-eye conditions, each session lasting 5 min. During the open-eye condition, participants were instructed to relax while minimizing eye movements to ensure the quality and precision of the collected data.

In the experimental validation, we employed a cross-participant validation protocol to ensure data integrity and mitigate potential issues related to data leakage. Based on a 10-fold cross-validation strategy, we partitioned the dataset into 10 subsets. During each training iteration, the model was trained on 9 sets of healthy subjects and 9 sets of MDD patients, reserving 1 set of each for testing. This process was repeated 10 times, rotating through each subset as the test set, and the average of these 10 test results was computed as the overall model performance. Additionally, to further enhance the reliability of the evaluation results, we conducted 3 random repetitions of the data-splitting process. At each time, the dataset was randomly partitioned into training and testing sets, and the model was assessed accordingly. The final performance evaluation was based on the average of these 3 random repetitions of testing results. This rigorous methodology ensures the model’s generalization across diverse data subsets and provides a stable assessment of its performance on unseen data. The corresponding experimental results are reported in Table 9. The results indicate that the proposed GraphEmotionNet model is also capable of classifying MDD patients from healthy patients. It shows that leveraging the nuanced patterns and deep-seated features extracted by GraphEmotionNet could expand its application to tasks such as identifying treatment responses, predicting relapse probabilities, and tailoring personalized therapeutic approaches for individuals with depressive conditions. The proposed GraphEmotionNet could aid in identifying early indicators or specific markers associated with different subtypes or stages of depression, thereby enabling more targeted and timely interventions.

**Table 9.** The accuracy results of the GraphEmotionNet model validation under closed-eye (EC) and open-eye (EO) conditions on the MDD database are presented

Session	First repetition	Second repetition	Third repetition	Average
EC	62.08 ± 10.08	62.39 ± 09.69	62.30 ± 08.34	62.25 ± 09.42
EO	62.24 ± 07.05	63.51 ± 09.77	65.17 ± 09.70	63.64 ± 09.01

## Conclusion

This paper introduces the GraphEmotionNet model, which addresses the incorporation of spatial–temporal information embedded within EEG signals in the context of dynamic emotional experiences. To overcome the constraints imposed by prior knowledge in graph construction, we introduce an adaptive graph learning approach to dynamically update graph structure to enhance the capture of more effective spatial–temporal feature representations. Furthermore, we incorporate domain adaptation to align the feature representations derived from different domains. This integration proves to be an effective approach for improving the model’s generalization capabilities. Through extensive experimental validations conducted on established databases, we verify the feasibility of the proposed GraphEmotionNet model. These experiments confirm the model’s potential to decode emotions using EEG signals under both within-subject and cross-subject scenarios in the field of affective brain–computer interface systems.

## Data Availability

This study utilized three public databases. SEED and SEED-IV can be consulted on the website https://bcmi.sjtu.edu.cn/home/seed/, while MDD can be accessed at https://figshare.com/articles/dataset/EEG_Data_New/4244171/2.
